# Utility of an Automated Artificial Intelligence Echocardiography Software in Risk Stratification of Hospitalized COVID-19 Patients

**DOI:** 10.3390/life12091413

**Published:** 2022-09-10

**Authors:** Tom Kai Ming Wang, Paul C. Cremer, Nicholas Chan, Hania Piotrowska, Gary Woodward, Wael A. Jaber

**Affiliations:** 1 Section of Cardiovascular Imaging, Heart, Vascular and Thoracic Institute, Cleveland Clinic, Cleveland, OH 44195, USA; 2 Ultromics Limited, University of Oxford, Oxford OX1 3PJ, UK

**Keywords:** echocardiography, COVID-19, artificial intelligence, machine learning, strain

## Abstract

Cardiovascular risk factors, biomarkers, and diseases are associated with poor prognosis in COVID-19 infections. Significant progress in artificial intelligence (AI) applied to cardiac imaging has recently been made. We assessed the utility of AI analytic software EchoGo in COVID-19 inpatients. Fifty consecutive COVID-19+ inpatients (age 66 ± 13 years, 22 women) who had echocardiography in 4/17/2020–8/5/2020 were analyzed with EchoGo software, with output correlated against standard echocardiography measurements. After adjustment for the APACHE-4 score, associations with clinical outcomes were assessed. Mean EchoGo outputs were left ventricular end-diastolic volume (LVEDV) 121 ± 42 mL, end-systolic volume (LVESV) 53 ± 30 mL, ejection fraction (LVEF) 58 ± 11%, and global longitudinal strain (GLS) −16.1 ± 5.1%. Pearson correlation coefficients (*p*-value) with standard measurements were 0.810 (<0.001), 0.873 (<0.001), 0.528 (<0.001), and 0.690 (<0.001). The primary endpoint occurred in 26 (52%) patients. Adjusting for APACHE-4 score, EchoGo LVEF and LVGLS were associated with the primary endpoint, odds ratios (95% confidence intervals) of 0.92 (0.85–0.99) and 1.22 (1.03–1.45) per 1% increase, respectively. Automated AI software is a new clinical tool that may assist with patient care. EchoGo LVEF and LVGLS were associated with adverse outcomes in hospitalized COVID-19 patients and can play a role in their risk stratification.

## 1. Introduction

COVID-19 infection caused by the severe acute respiratory syndrome-related coronavirus 2 has become one of the most devastating global pandemics in history. Recent studies have identified that many cardiovascular diseases, cardiovascular risk factors, elevated cardiac biomarkers signaling acute myocardial injury, and other cardiac manifestations to be associated with worse prognosis in patients with COVID-19 infection [1]. Transthoracic echocardiography (TTE) remains the first-line cardiac imaging modality for cardiac assessment in COVID-19 infection, and societal guidelines have been published with regards to when and how to perform TTE safely in patients with COVID-19 infection [2]. Some studies have demonstrated TTE’s clinical value for diagnosis and potentially altering subsequent management of patients with COVID-19 infection [3]. Significant progress has been made in the field of artificial intelligence (AI) and machine learning over the last decade when applied to multi-modality cardiac imaging, including for TTE [4]. AI techniques have the potential for rapid and streamlined analysis of point-of-care and complete TTE examinations such as inpatients with COVID-19 infection, for which, in the current ongoing pandemic, there remains unmet clinical need. It may also facilitate risk stratification, however, so far, this has been infrequently studied in this setting [5].

In this cohort study of COVID-19 inpatients, we aimed to compare the automated AI analytic software “EchoGo” (Ultromics Limited, Oxford) output with standard TTE measurements of the left ventricle and assess their prognostic utility for adverse in-hospital outcomes.

## 2. Materials and Methods

Consecutive patients with COVID-19 infection confirmed on a polymerase chain reaction test at 18 years of age or older who underwent inpatient clinically indicated TTE during 17 April 2020–8 May 2020 at Cleveland Clinic were identified from the prospective COVID-19 inpatient registry of our institution (*n* = 80). Exclusion criteria included TTE studies without apical 4- and/or 2-chamber views to allow for EchoGo analysis (*n* = 7), and suboptimal image quality, which the EchoGo software could not analyze to produce an output (*n* = 23), leaving 50 patients included in the study. Relevant demographic, past history, and admission laboratory tests data were collected for electronic medical records. History of coronary heart disease was defined as at least one coronary artery with ≥50% stenosis, percutaneous coronary intervention, coronary artery bypass grafting, or prior myocardial infarction. Valvular heart disease requires the presence at least one heart valve lesion (stenosis or regurgitation) graded moderate or severe. Laboratory test values on admission were recorded. The Acute Physiology and Chronic Health Evaluation IV (APACHE-IV) score was retrospectively calculated.

All TTEs were performed at the bedside with appropriate protective equipment with a limited targeted protocol, as opposed to the standard comprehensive TTE assessment. TTE was performed using Philips EPIQ 7C (Philips Medical Systems, Bothell, WA, USA) or GE Vivid7 or Vivid9 (GE Medical, Milwaukee, WI, USA). EchoGo is the automated machine learning software used to determine automated left ventricular end-diastolic volume (LVEDV), end-systolic volume (LVESV), ejection fraction (LVEF), and global longitudinal strain (LVGLS) measurements, using the apical 4-chamber and 2-chamber views. Standard measurements of these left ventricle parameters were also performed using the Simpson’s biplane technique for LVEF and velocity-vector imaging technique for LVGLS, with the latter being a vendor neutral platform to analyze both Philips and GE TEE images. Pericardial effusion was counted if at least small in size. The primary endpoint was the composite of in-hospital all-cause death, acute myocardial injury (defined as rise and/or fall in troponin biomarkers above the upper reference limit), and need for mechanical ventilation.

Data was presented as mean ± standard deviation for continuous variables and frequency (percentage) for categorical variables. Correlations between EchoGo and standard measurements of the left ventricle were analyzed using Deming regression, Pearson correlation coefficient (r), and Bland–Altman plots. Multivariable analyses were performed using logistic regression of the primary endpoint with each echocardiography parameter, adjusted for the APACHE-IV score (to condense clinical parameters given the limited number of endpoints) [6]. Statistical analyses were performed using R (version 4.0.3, R Foundation for Statistical Computing, Vienna, Austria) and Prism (version 8, GraphPad, San Diego, CA, USA). Institutional Board Review approval was obtained at the beginning of this study from our institution’s IRB office with a patient consent waiver. The data that support the findings of this study are available on reasonable request from the corresponding author.

## 3. Results

Key cohort characteristics, including clinical, biomarker, and echocardiography factors, are shown in Table 1. Amongst the 50 patients with COVID-19 infection studied, the mean age was 66 ± 13 years, 22 (44%) were women, and the mean body mass index was 30 ± 7 kg/m^2^. Past history included coronary heart disease in 16 (32%), heart failure hospitalization in 20 (40%), hypertension in 42 (84%), diabetes in 26 (52%), atrial fibrillation in 9 (18%), stroke in 7 (14%), and chronic respiratory disease in 22 (44%). Laboratory tests included mean creatinine of 2.0 ± 1.6 mg/dL, C-reactive protein of 10.9 ± 6.4 mg/L, and high-sensitivity troponin T of 75 ± 129 ng/dL. The mean APACHE-IV score was 59 ± 23. The primary endpoint occurred in 24 (48%) patients, including 8 (16%) deaths, 21 (42%) acute myocardial injuries, and 16 (32%) needing mechanical ventilation while the mean length of hospital stay was 14 ± 10 days.

**Figure 1:**
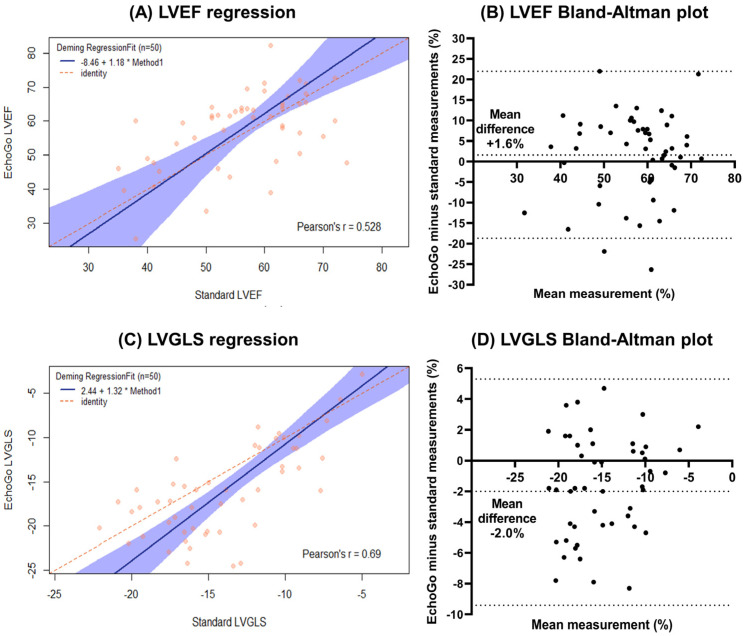
EchoGo software versus standard echocardiography measurements for left ventricular ejection fraction (LVEF) and left ventricular global longitudinal systolic strain (LVGLS)—(**A**) Deming regression plot with Pearson correlation coefficients for LVEF; (**B**) Bland–Altman plots for LVEF; (**C**) Deming regression plot with Pearson correlation coefficients for LVGLS and (**A**,**D**) Bland–Altman plots for LVGLS.

**Figure 2:**
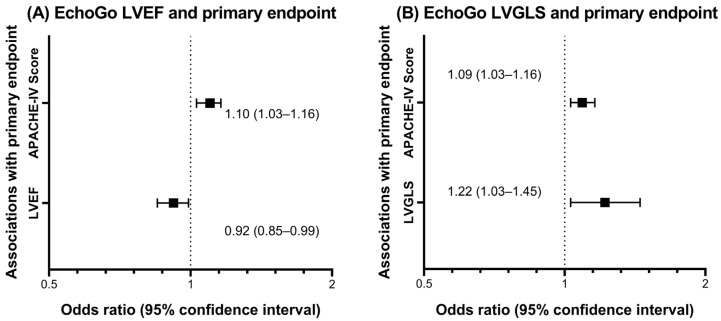
Logistic regression analysis of the primary endpoint (composite of in-hospital all-cause death, acute myocardial injury, and need for mechanical ventilation) in COVID-19 inpatients for EchoGo measured (**A**) left ventricular ejection fraction (LVEF) and (**B**) left ventricular global longitudinal strain (LVGLS). Data presented as Forest plots of the odds ratios (95% confidence intervals) in the multivariable model adjusted for the Acute Physiology and Chronic Health Evaluation IV score (APACHE-IV) score.

By standard TTE and EchoGo measurements, LVEDV were 120 ± 41 and 121 ± 42 mL, LVESV 54 ± 28 and 53 ± 30 mL, LVEF 56 ± 10 and 58 ± 11%, and LVGLS −14.1 ± 4.2 and −16.1 ± 5.1%, respectively. Comparing these two methods of left ventricular measurements, there was high correlation for LVEDV (r = 0.81) and LVESV (r = 0.87), and moderate correlation for LVEF (r = 0.53) and LVGLS (r = 0.69), all with *p*-value < 0.001. The Deming regression and Bland–Altman plots are shown in Figure 1 for LVEF and LVGLS. Adjusted for the APACHE-IV score, EchoGo LVEF and LVGLS were independently associated with the primary endpoint, odds ratios (95% confidence intervals) of 0.92 (0.85–0.99) and 1.22 (1.03–1.45) per 1% increase, respectively, as indicated in the Forest plots of Figure 2, while the other EchoGo and standard TTE measurements were not associated with the primary endpoint.

## 4. Discussion

Many studies since the pandemic have found cardiovascular co-morbidities and cardiac injury to portend worse prognosis in COVID-19 patients, which makes TTE critical in the cardiac evaluation of these patients [1]. This study is one of the first to evaluate AI TTE software in patients with COVID-19, along with the recent World Alliance Societies of Echocardiography (WASE-COVID) study, and has several important findings [5]. EchoGo AI software had high LVEDV and LVESV and moderate LVEF and LVGLS correlations with standard TTE measurements in our COVID-19 inpatients cohort, confirming its utility for left ventricular measurements in bedside TTE and targeted protocols as an alternate method. The lower correlations for LVEF and LVGLS may be because of the discrepancies in the volumes being compounded in the calculation of LVEF, along with known variations in strain values that differ amongst the measurement software vendors.

Uniquely, we found that EchoGo LVEF and LVGLS values both provided incremental prognostic value to the well-established APACHE-IV score in COVID-19 inpatients, whereas standard TTE measurements did not. We suspect one reason could be the suboptimal quality of some of the bedside TTE studies for COVID-19 inpatients, which made standard measurements more challenging and potentially less accurate and reproducible than EchoGo AI software automated measurements so that only the latter demonstrated prognostic value. These findings, together with the efficiency, automated, and potential for batch analysis of TTE scans are all strengths of the EchoGo AI software. The WASE-COVID study similarly found LVGLS (using EchoGo software) and right ventricular free wall longitudinal strain (using TomTec software) but not LVEF (standard measurement), as the TTE parameters independently associated with in-hospital mortality in patients with COVID-19 infections, with other adverse prognosticators being older age, previous lung disease, and increased lactic dehydrogenase [5]. This study did not, however, compare and correlate EchoGo and standard measurements of the left ventricular volumes and function of these patients. Taken together, the findings support the uptake of automated AI software such as EchoGo for left ventricular analysis in COVID-19 given its prognostic value, accuracy, and efficiency in TTE analyses to potentially aid patient management, and may be expanded to many other cardiovascular diseases as well, subject to further research. There are also other roles for AI and machine learning in COVID-19 management and research, whether for interpretation of other medical tests, construction of diagnostic and treatment algorithms, and development of population screening and preventative (including vaccination) strategies [7,8,9].

This study has some limitations of note. It is a single center observational cohort study with inherent biases. Study power and multivariable analyses are restrained by the number of patients and clinical events, so the APACHE-IV score was used as a surrogate to measure global clinical risk. A minority of patients were excluded because of suboptimal image quality, which is to be expected for bedside TTE studies of sick COVID-19 patients, some of whom were in the intensive care unit. The EchoGo software currently only analyzes a limited number of TTE parameters, although there is ongoing software development to expand its analytic capabilities. The Velocity Vector Imaging technique was used for standard strain measurement analysis as it is a vendor neutral method, although it is known to have a slightly lower magnitude of LVGLS than other vendors such as GE EchoPAC and may also have explained its slightly lower LVGLS values than EchoGo. We also focused on assessing associations between in-hospital outcomes and TTE, including EchoGo measurements, rather than longer-term outcomes beyond hospital discharge, where further research is warranted.

In conclusion, automated AI software is a new clinical tool that may assist with patient care, with potential for higher efficiency and precision of cardiac imaging analysis, including in acutely ill patients with COVID-19 infection. EchoGo software output had high correlations with left ventricular volumes and moderate correlations with LVEF and LVGLS compared with standard TTE measurements. EchoGo LVEF and LVGLS measurements were associated with the primary endpoint, including when adjusted for the APACHE-IV score, and can therefore play a role in the risk stratification of hospitalized COVID-19 patients.

## Figures and Tables

**Table 1:** Clinical, biomarker, and echocardiography characteristics of the COVID-19 inpatients cohort.

Number of Patients	50
**Demographics/presentation**	
Age (years)	66 ± 13
Female	22 (44%)
Body mass index (kg/m^2^)	30 ± 7
Body surface area (m^2^)	2.03 ± 0.28
APACHE-4 * score	59 ± 23
**Past history**	
Coronary heart disease	16 (32%)
Heart failure	20 (40%)
Valve disease **	5 (10%)
Cardiac surgery	7 (14%)
Cardiac implantable electronic device	2 (4%)
Atrial fibrillation	9 (18%)
Hypertension	42 (84%)
Hyperlipidemia	16 (32%)
Diabetes	26 (52%)
Current smoker	4 (8%)
Past smoker	18 (36%)
Stroke	7 (14%)
Chronic respiratory disease	22 (44%)
Dialysis	5 (10%)
Active malignancy	5 (10%)
Solid organ transplant	4 (8%)
**Laboratory tests on admission**	
Creatinine (mg/dL)	2.0 ± 1.6
Hs-Troponin T (ng/L)	75 ± 129
C-reactive protein (mg/L)	10.9 ± 6.4
Procalcitonin (ng/dL)	1.4 ± 2.2
Albumin (g/dL)	3.5 ± 0.5
Hemoglobin (g/dL)	12.1 ± 2.3
**Echocardiography (standard measurements)**	
Left ventricle end diastolic volume (mL)	120 ± 41
Left ventricle end systolic volume (mL)	54 ± 28
Left ventricle ejection fraction (%)	56 ± 10
Left ventricular global longitudinal strain (%)	−14.1 ± 4.2%
Right ventricle dilation	6 (12%)
Right ventricle systolic impairment	11 (22%)
Tricuspid annular peak systolic excursion (mm)	17 ± 7
Right ventricle systolic tissue doppler (mm/s)	10.7 ± 2.4
Aortic regurgitation **	2 (4%)
Aortic stenosis **	1 (2%)
Mitral regurgitation **	5 (10%)
Tricuspid regurgitation **	8 (16%)
Pulmonary hypertension	12 (24%)
Pericardial effusion ***	3 (6%)
EchoGo measurements	
Left ventricle end diastolic volume (mL)	121 ± 42
Left ventricle end systolic volume (mL)	53 ± 30
Left ventricle ejection fraction (%)	58 ± 11
Left ventricle global longitudinal strain (%)	−16.1 ± 5.1%

Data is presented as mean ± standard deviation for continuous parameters and frequency (percentage) for categorical variables; * APACHE-IV = Acute Physiology and Chronic Health Evaluation IV score; ** Valve disease and lesions counted only if at least moderate in severity; *** Pericardial effusion counted if at least small in size.

## Data Availability

The data that support the findings of this study are available on reasonable request from the corresponding author.
